# Back Pain: A Rare Presentation of Takayasu Arteritis

**DOI:** 10.7759/cureus.4028

**Published:** 2019-02-07

**Authors:** Hussain Azhar, Syed Asad Hasan Rizvi, Amna A Siddiqui, Fatima Q Siddiqui

**Affiliations:** 1 Internal Medicine, Civil Hospital Karachi, Karachi, PAK; 2 Internal Hospital, Civil Hospital Karachi, Karachi, PAK

**Keywords:** vasculitis, takayasu arteritis, back pain, aorta, case report

## Abstract

Takayasu arteritis (TA) is a chronic inflammatory large-vessel vasculitis of the aorta and its major branches. It is a relatively rare disease, which presents with a wide spectrum of clinical features. Back pain is, however, rarely described to be a presenting symptom of TA. We report a case of a 28-year-old female with no known co-morbidity, who presented with back pain along with intermittent fever, dry cough, and significant weight loss. After an extensive inpatient workup, a computed tomography (CT) scan with contrast of chest and abdomen revealed evidence of vasculitis involving aorta, common carotid arteries, renal arteries, common iliac arteries as well as external and internal iliac arteries. A confirmatory CT abdominal aortography established the diagnosis of TA. The patient was treated with long-term oral corticosteroid therapy. Our case highlights that while assessing nonspecific back pain with elevated inflammatory parameters, particularly in young women, TA should be considered as one of the differential diagnoses.

## Introduction

First described by Mikito Takayasu in 1908 [1], Takayasu arteritis (TA) is a chronic granulomatous large-vessel vasculitis that predominantly involves the aorta and its major branch arteries. It may also involve the coronary and the pulmonary arteries. It is typically seen in young women of Asian descent. TA is a rare disease, with the highest ever annual mean incidence and prevalence rates of 0.24 and 2.82 per hundred thousand, respectively, reported in Korea [2]. It may present with a wide variety of clinical features ranging from early nonspecific constitutional symptoms such as fever, weight loss, and malaise to late life-threatening cardiovascular and neurological complications [3]. Musculoskeletal symptoms are also common including arthralgia and myalgia. Back pain, however, is a rarely reported clinical presentation of TA. We report a case of a 28-year-old female who presented with mid-dorsal back pain and was later diagnosed with TA.

## Case presentation

A 28-year-old female with no known co-morbidity was admitted to a tertiary care hospital in Karachi in March 2018 with a history of mid-dorsal back pain and moderate intermittent fever for five months. She also complained of a dry cough and significant weight loss of about 10 kg for the past two months. The patient described the back pain as gradual in onset, moderate in intensity, nonradiating, and dull in character. It aggravated with physical activity and improved with rest and the use of non-steroidal anti-inflammatory drugs (NSAIDs). It was associated with arthralgia of small joints of hands, wrists, and ankles. The patient denied having any morning stiffness but complained of worsening fatigue throughout the day. On physical examination, she had a blood pressure of 110/80 mmHg, a temperature of 101°F, a pulse rate of 106/min, and a respiratory rate of 18/min. Pallor of skin and conjunctiva was present. All peripheral pulses were palpable with regular rhythm but low volume. No radio-radial, radio-femoral delay or difference in blood pressure of the upper extremities was noted. No bruit was audible on neck, chest, or abdomen. Mild tenderness over mid-dorsal spine at D7-8 vertebral and paravertebral region was found. There was a full but slightly painful range of motion of the spine. Findings of examination of all other systems, including gynecological and obstetrics examination, were unremarkable.

Laboratory investigations revealed normocytic normochromic anemia with hemoglobin of 9.2 g/dL and hematocrit of 28.9%. Total leukocyte count (TLC) was 9,600 cells/μL with 83% neutrophils and 13% lymphocytes. Erythrocyte sedimentation rate (ESR) was elevated on repeated tests with the latest reports showing levels of 135 mm/h, while C-reactive protein (CRP) levels were normal. Total serum proteins and serum albumin levels were within normal range. Serum globulin level was raised (4.9 g/dL), while albumin to globulin ratio (A/G) was slightly below normal values (0.6). The results for renal function tests, liver function tests, coagulation profile, uric acid, and angiotensin-converting enzyme (ACE) levels were all within normal range. Tests for autoimmune markers including anti-nuclear antibody (ANA), anti-smooth muscle antibody (ASMA), anti-mitochondrial antibody (AMA), anti-cyclic citrullinated peptide (anti-CCP), and rheumatoid factor (RF) were all negative. Blood cultures were also negative. Upon imaging, chest X-ray, echocardiography, and ultrasound abdomen and pelvis showed no abnormalities. X-ray and magnetic resonance imaging (MRI) with contrast of dorso-lumbar spine were also unremarkable.

**Figure 1:**
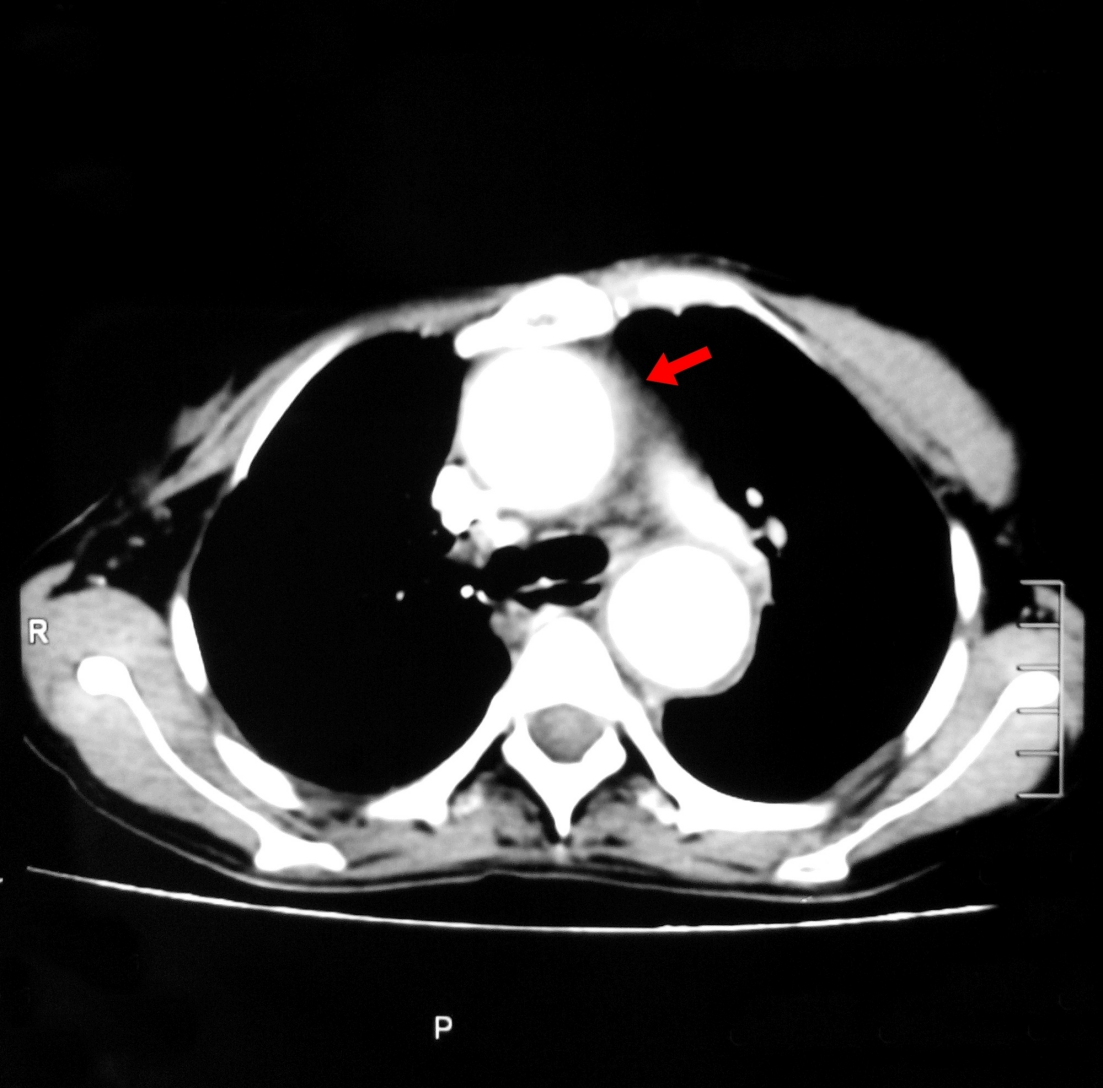
Computed tomography (CT) scan with contrast of chest showing dilation of aorta.

After a week of extensive inpatient workup with no identifiable cause of fever, the case was labeled as pyrexia of unknown origin. Considering a long-standing history of back pain, fever, weight loss, and an elevated ESR in a tuberculosis (TB) endemic region, there was high suspicion of extra-pulmonary TB. To evaluate for extra-pulmonary TB, and also for vasculitis, possible abscess, and hidden malignancy, a computed tomography (CT) scan with contrast of chest and abdomen was performed. As shown in Figure 1, it revealed diffuse intimal thickening and dilation in ascending aorta, aortic arch, descending thoracic, and abdominal aorta. Superiorly, the lesion involved both common carotid arteries. Inferiorly, it involved bilateral renal arteries with extension into both common iliac, external and internal iliac arteries. Dissecting intimal flap was seen at the junction of thoracoabdominal aorta. Furthermore, multiple infarcts were noted in the upper lobe of the right lung along with nodularity and septal thickening in the lingular segment. These findings were suggestive of vasculitis.

**Figure 2:**
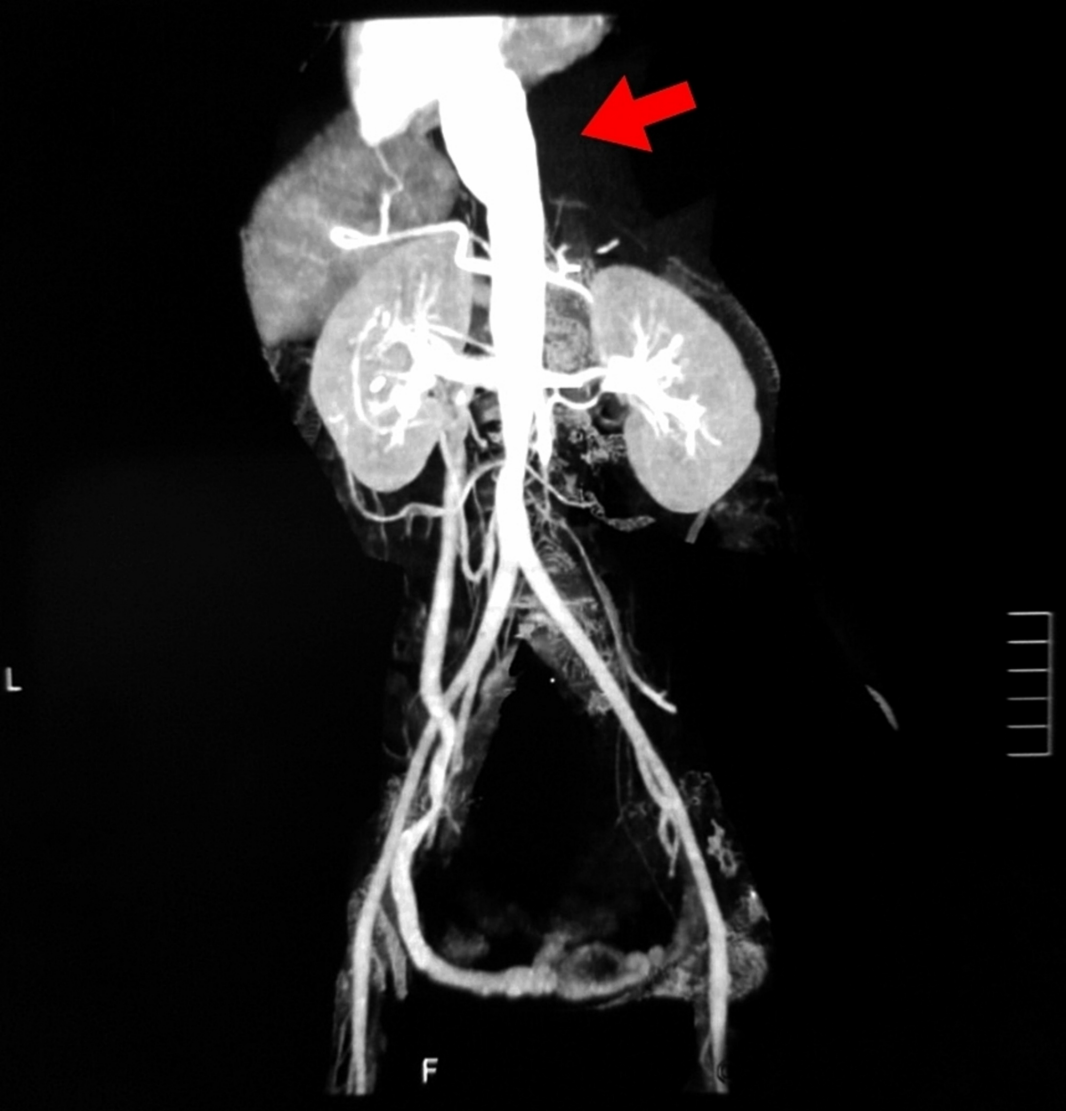
Abdominal aortography showing focal fusiform dilation of the abdominal aorta.

Assuming a diagnosis of vasculitis, a confirmatory CT abdominal aortography was performed which similarly revealed diffuse intimal thickening of thoracoabdominal aorta along with its focal fusiform dilation and an intimal dip as shown in Figure 2. Multiple focal intimal thickening and narrowing were noted in superior mesenteric and bilateral renal arteries. Origin of celiac trunk was narrowed. Based on these findings, a final diagnosis of TA type five with aortic dissection and vasculitic pulmonary infarcts was made.

The patient was treated with long-term oral corticosteroid therapy with a dosage of 1 mg/kg daily, which led to a resolution of back pain and fever and decline in ESR levels. Azathioprine was added to sustain remission. The patient is being followed up and remains in remission till date.

## Discussion

Takayasu arteritis is an idiopathic progressive inflammatory disease of the aorta and its branch arteries. TA has a complex clinical course, which is simplified by classification into three stages for the purpose of understanding [4]. The first stage of the disease is the pre-vasculitic systemic stage, during which the patient presents with nonspecific constitutional symptoms like malaise, fever, night sweats, arthralgia, headache, anorexia, and weight loss [5]. The second stage is the vascular inflammatory stage, which is characterized by arterial injury and pain with stenosis, aneurysm, and occlusion [4]. This stage can manifest with diminished or absent pulses (mainly in radial arteries), vascular bruits, renovascular hypertension (abdominal aorta involvement), chronic mesenteric ischemia, retinopathy, aortic regurgitation (ascending aorta involvement), myocardial infarction, limb claudication (distal aortic involvement), and neurological symptoms like orthostatic hypotension, dizziness, seizures, transient ischemic attacks, stroke, hemiplegia, and paraplegia [5]. Carotidynia may also occur during this stage [6]. The third stage is the burnt-out fibrotic stage, which is characterized by minimal symptoms and remission of the disease [4]. It is, however, important to understand that the constitutional and vascular symptoms may occur at the same time. In addition, the stages are difficult to define clinically, and that the systemic inflammatory stage may even be absent [4]. The practical implication of this classification is, therefore, limited.

Although a wide variety of heterogeneous symptoms have been reported, backache is rarely described in the medical literature as the presenting symptom of TA. Melamed et al. [7] reported a case of a young woman presenting with acute-onset thoracic back pain, fever, and fatigue. This back pain was eventually diagnosed as a manifestation of TA based on the CT angiogram findings. There was a previous history of ulcerative colitis in this patient, which is known to be associated with TA [5]. This association was, however, absent in our case. Shikino et al. [8] described the clinical characteristics of a patient who presented with fever and thoracic back pain, later diagnosed as a case of TA by imaging. In this case, epigastric palpation had revealed pulsatile tenderness due to abdominal aorta involvement. In contrast, no epigastric tenderness was found in our case. Noyer et al. [9] reported a case of a young boy with a history of tuberculosis and sickle cell disease who presented with dorsal back pain. It was associated with the inflammatory syndrome, coarctation of the aorta, stenosis of the renal arteries, along with hypertension and asymmetrical blood pressure between the upper and the lower limbs. Diagnosis of TA was made based on clinical findings and imaging. Another case of TA was described by Fanning and Hickey [10], in which the patient presented with the complaints of persistent lower back pain, drop attacks, asthenia, unexplained weight loss, intermittent fever, dizziness, and lower limb claudication. There was a difference in blood pressure between the right and left arms, and arterial bruits were also present. Similarly, Slobodin et al. [11] presented a case of TA, in which the patient presented with a poorly localized pain in the thoracic and lumbar spine. Brachial and radial pulses were absent, while arterial bruits were audible. In contrast to all of these cases, our case presented with vague nonspecific signs and symptoms with no known associated conditions, which made the final diagnosis difficult. As TA causes injury to the aorta and its branches, back pain, although less acknowledged, can present as the initial complaint. Slobodin et al. postulated that back pain may occur due to the irritation of the nerves present in the adventitia-media junction following the inflammation of the adventitia. Mechanoreceptors and chemical nociceptors may have a role behind the pain caused by the thickened, inflamed aorta due to decreased compliance and release of cytokines [11].

Takayasu arteritis remains a diagnostic challenge. Due to the low specificity of laboratory investigations and the lack of feasibility of arterial biopsies, the diagnosis of TA depends upon clinical assessment and imaging [5]. Its diagnosis may be guided by modified Ishikawa’s criteria [12]. Diagnostic criteria and imaging modalities with the ability to identify early-stage systemic disease are, however, still lacking [13]. Among the imaging modalities that can be used, CT angiography is one option which has 95% sensitivity and 100% specificity (using catheter-based angiography as the gold standard) [13]. In our case, CT angiography was successful in establishing the diagnosis. Imaging aside, the diagnosis of TA principally depends upon the decision of a physician to include the disease in his differential diagnoses [6]. A conclusive diagnosis is often delayed, possibly due to the rarity of the disease and widely variable disease presentation [6]. The lack of acknowledgment of back pain as a presenting complaint of TA can lead to further failure of early diagnosis and management. We, therefore, recommend that physicians consider TA as a differential diagnosis while assessing nonspecific back pain with elevated inflammatory parameters, particularly in young women.

## Conclusions

Our case highlights the importance of considering back pain as a presenting complaint of a patient with TA.
